# Facile Synthesis of Rhodium Nanodendrites with Enhanced Activity toward Hydrazine-Assisted Water Splitting

**DOI:** 10.1021/acs.chemmater.6c00125

**Published:** 2026-04-21

**Authors:** Jiaqi Guan, Zhiqi Wang, Kei Kwan Li, Yong Ding, Younan Xia

**Affiliations:** † School of Chemistry and Biochemistry, 1466 Georgia Institute of Technology, Atlanta, Georgia 30332, United States; ‡ School of Materials Science and Engineering, 256693 Georgia Institute of Technology, Atlanta, Georgia 30332, United States; § The Wallace H. Coulter Department of Biomedical Engineering, Georgia Institute of Technology and Emory University, Atlanta, Georgia 30332, United States; ∥ Department of Materials Science and Engineering, Department of Biomedical Engineering, Johns Hopkins University, Baltimore, Maryland 21218, United States

## Abstract

We report an aqueous synthesis of Rhodium (Rh) nanodendrites and evaluation of their merit as a bifunctional electrocatalyst toward hydrazine-assisted water splitting. The formation of dendritic morphology can be attributed to the fast reduction kinetics responsible for burst nucleation and then attachment growth. Rapid reduction results in a high concentration of Rh atoms, which quickly nucleate and grow into ultrafine Rh nanocrystals, followed by their evolution into dendritic structures through attachment growth. Extending the reaction time naturally prolongs the number of branched arms while increasing the overall size of the particles. Aging the Rh­(III) precursor solution slows down the reduction kinetics, leading to the formation of fewer Rh nanoparticles for the generation of smaller dendrites. The reaction temperature influences the reduction kinetics during nucleation and then aggregation thermodynamics governing the attachment process. Together with a high specific surface area, the lattice defects arising from attachment growth induce tensile strain, resulting in more and better catalytic sites. When evaluated as a bifunctional electrocatalyst toward hydrazine oxidation and hydrogen evolution reactions, the Rh nanodendrites gave an enhanced mass activity of 162.0 A mg^–1^ at 0.20 V vs RHE for hydrazine oxidation and 8.6 A mg^–1^ at −0.07 V vs RHE for hydrogen evolution, approximately 1.5 times greater than that of 5 nm Rh nanocubes, highlighting their promise for applications such as hydrazine-assisted water splitting.

## Graphical abstract

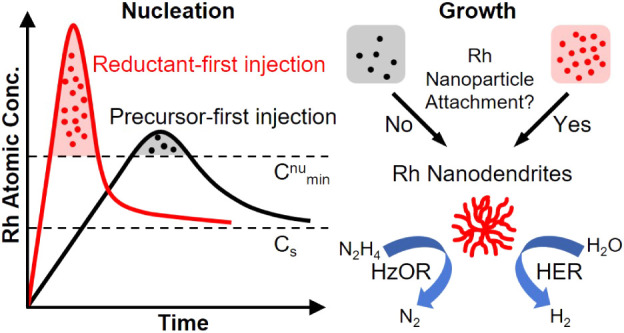

## Introduction

Dendritic metal nanostructures have emerged as a unique class of electrocatalytic materials owing to their highly branched morphology.^1–3^ Compared with conventional nanoparticles, dendritic nanostructures are known to possess abundant active sites such as tips, edges, and high-index facets while offering markedly larger electrochemically active surface areas (ECSAs).^4–7^ Moreover, since nanodendrites are typically formed through the attachment growth mechanism,^8,9^ attachments involving large misorientation angles between colliding nanoparticles are unavoidable, leading to a polycrystalline structure rich in defects such as twin boundaries.^10,11^ Altogether, the large specific surface area and high density of undercoordinated atoms, together with defect-induced lattice distortions such as tensile strain, can effectively modulate the electronic structure and optimize the adsorption energy of intermediates in a catalytic reaction, enabling a more favorable reaction pathway and lowering the associated reaction energy barrier.^12–14^ As a result, metallic nanodendrites have been demonstrated with superior catalytic performance in a broad range of electrochemical reactions.^15–17^

Conventional alkaline water electrolysis, although widely adopted for hydrogen production, is fundamentally limited by the sluggish oxygen evolution reaction (OER), which requires a large overpotential.^18,19^ In contrast, hydrazine-assisted water splitting provides a thermodynamically advantageous alternative. The hydrazine oxidation reaction (HzOR) exhibits a much lower electrochemical potential and faster kinetics than OER, initiating hydrogen production at dramatically reduced cell voltages while simultaneously enabling the degradation of hydrazine as a toxic component in industrial wastewater.^20,21^ Rhodium (Rh) is particularly attractive as a catalytic material for the hydrazine-assisted electrocatalytic systems due to its intrinsically high activity toward both HzOR and the hydrogen evolution reaction (HER) and strong resistance to oxidation.^22–24^

Fabrication of Rh nanostructures with maximized surface accessibility and strain-modulated active sites is a promising strategy for developing efficient bifunctional catalysts toward HzOR and HER. Despite their advantages, the synthesis of Rh nanostructures with a dendritic morphology remains to be further explored. Current synthetic protocols for Rh nanodendrites often require demanding environments, such as elevated temperatures in an oil phase,^25^ complex templates,^26^ or halide additives such as F^–^ or I^–^,^27,28^ to induce voids and branches. Specifically, Feng et al. synthesized Rh nanodendrites by heating Rh­(acac)_3_ in oleylamine at 160 °C for 2 h under N_2_. Jiang et al. used PEO-*b*-PMMA polymeric micelles in a mixture of dimethylformamide (DMF) and water to prepare mesoporous Rh by heating the system at 60 °C for 12 h, and the soft template was found to be essential to the construction of pores. Yuan et al. reported that Rh nanodendrites could be prepared in an aqueous system, but the synthesis required sodium lauryl sulfate (SLS) and F^–^ ions as additives and hydrothermal conditions at 220 °C for 6 h. Zou et al. also employed an iodide-mediated templating strategy in an aqueous cetyltrimethylammonium bromide (CTAB) solution at 80 °C for 3 h. These two studies suggested that control of morphology in an aqueous medium critically depended on the assistance of additives such as F^–^ and I^–^ ions. The oil-phase synthesis typically requires elevated energy consumption, extended reaction time, and complicated postsynthetic purification, limiting scalability and utility. On the other hand, template-assisted and additive-dependent strategies not only increase the complexity but also obscure the growth mechanism of dendritic formation. In general, the fundamental mechanism governing Rh nanodendrite formation remains poorly understood, particularly regarding the impacts of specific parameters on the nucleation and attachment growth of ultrafine Rh nanoparticles.

In this work, we establish a facile and robust aqueous route to the synthesis of rhodium dendritic nanostructures at relatively low temperatures. By controlling the sequence of reagent addition, precursor coordination state, reaction time, and temperature, we elucidate the mechanistic details involved in the nucleation, attachment growth, and morphological development during the formation of Rh nanodendrites. The resulting dendrites feature ultrathin branches and pervasive twin boundaries as well as substantial tensile strain originating from the attachment of primary nanoparticles. We further demonstrate that these structural characteristics endow the Rh nanodendrites with superior bifunctional catalytic performance toward both the HzOR and HER. This work provides not only an accessible synthetic platform for Rh nanodendrites but also mechanistic insights into their catalytic behavior.

## Experimental Section

### Chemicals and Materials

Sodium hexachlororhodate­(III) (Na_3_RhCl_6_, 97%), sodium borohydride (NaBH_4_, 98.0%), l-ascorbic acid (AA, 99%), poly­(vinylpyrrolidone) (PVP, M_W_ ≈ 55,000), cetyltrimethylammonium chloride (CTAC, 25 wt % in water), cetyltrimethylammonium bromide (CTAB, ≥99.0%), sodium citrate tribasic dihydrate (Na_3_C_6_H_5_O_7_ · 2H_2_O, ≥99.0%), potassium bromide (KBr, ≥99%), potassium hydroxide (KOH, ≥85%), and hydrazine (N_2_H_4_, 98%) were all obtained from Sigma-Aldrich and used as received. Ethylene glycol (EG, ≥99%) was purchased from J. T. Baker. Deionized (DI) water with a resistivity of 18.2 MΩ·cm at room temperature was used throughout the experiments.

### Characterizations

A Hitachi HT7700 microscope was used at 120 kV to take transmission electron microscopy (TEM) images. Ultraviolet–visible (UV–vis) spectra were obtained on a Cary 60 spectrometer (Agilent Technologies, Santa Clara, CA). The Rh compositions of inks prepared from Rh dendrites and Rh nanocubes were determined through the use of an inductively coupled plasma mass spectrometer (ICP-MS, NexION 300Q, PerkinElmer, Waltham, MA). High-resolution transmission electron microscopy (HRTEM) analyses were conducted using an FEI Tecnai G2 F30 TEM instrument operated at 200 kV. High-angle annular dark-field scanning transmission electron microscopy (HAADF-STEM) images were acquired using a Hitachi HD-2700 microscope at 200 kV and a ThermoFisher Spectra 300 S/TEM. X-ray diffraction (XRD) patterns were obtained by using Rigaku Miniflex Powder XRD. X-ray photoelectron spectroscopy (XPS) analyses were conducted using a Thermo K-Alpha (Thermo Fisher Scientific, Waltham, MA, USA).

### Synthesis of Rh Dendrites

In a standard synthesis, 2 mL of aqueous CTAC (100 mM) and 130 μL of aqueous NaBH_4_ (100 mM) were mixed in a 20 mL glass vial under magnetic stirring at a speed of 600 rpm and at 90 °C. After about 5 s, 200 μL of an aqueous Na_3_RhCl_6_ solution (2.0, 5.0, or 10.0 mM), freshly prepared or aged in air at 25 °C for 3 weeks, was injected in one shot, and the reaction was allowed to proceed at 90 °C for 30 min, 60 min, and 4 h. The solid products were collected by centrifugation at 17500 rpm for 40 min and washed twice with deionized water. A 10-fold scale-up synthesis was carried out in a 50 mL three-neck flask under the same procedure above.

### Synthesis of Rh Quasi-Spherical Particles

In a typical synthesis, 2 mL of aqueous CTAC (100 mM) and 200 μL of aqueous Na_3_RhCl_6_ (10.0 mM) were mixed in a 20 mL glass vial under magnetic stirring at a speed of 600 rpm and at 90 °C. After about 5 s, 130 μL of aqueous NaBH_4_ (100 mM) was injected in one shot, and the reaction was allowed to proceed at 90 °C for 30 min and 12 h. The solid products were collected by centrifugation at 17500 rpm for 50 min and washed twice with deionized water.

### Synthesis of 5 nm Rh Nanocubes

The synthesis was conducted by following a previous work from our group.^29^ Specifically, 13 mL of an EG solution containing AA (52.8 mg), KBr (108 mg), and PVP (133 mg) was transferred into a three-neck flask and heated at 140 °C under magnetic stirring (380 rpm) for 1 h. Meanwhile, 6 mL of another EG solution containing 46.2 mg of Na_3_RhCl_6_ was added into the flask at a rate of 60 mL·h^–1^ for the first 1.1 mL and 4 mL·h^–1^ for the remaining 4.9 mL, respectively. After 3 h, the solid products were collected by centrifugation, washed once with acetone and three times with a mixture of ethanol and acetone at a ratio of 3:1, and dispersed in deionized water for further use.

### Electrochemical Measurements

All electrochemical measurements were performed at room temperature in a three-electrode cell connected with a CHI 600E electrochemical workstation (CHI 600E, CH Instruments, USA). For the electrochemical activity test, a Pt wire served as the counter electrode, together with a single junction Hg/HgO reference electrode (+98 mV vs NHE, PINE Research). A glassy carbon (GC) electrode (0.196 cm^2^ area) served as the working electrode. Catalyst inks were prepared by dispersing 80 μL of the carbon-supported catalyst suspension with 4 μL of Nafion (5 wt %, VWR) in a 1.5 mL centrifuge tube, followed by sonication in an ice bath for 2 h. Subsequently, 5 μL of the ink was drop-cast onto the GC electrode and dried in air. The actual Rh loading of the catalyst was 1.0 μg/cm^2^ determined by ICP-MS analysis, and electrochemical results were normalized to the mass of the catalyst. All potentials were measured versus Hg/HgO and then converted to values relative to the reversible hydrogen electrode (RHE) according to *E* (V_RHE_) = *E* (V_Hg/HgO_) + 0.098 V + 0.0592 × pH. Prior to measurements, the GC electrode was cycled 12 times between 0.05 and 1.1 V vs RHE at 50 mV s^–1^ in an Ar-saturated KOH solution (1.0 M) until a stable cyclic voltammetry (CV) curve was obtained. The electrode was then measured in the linear sweep voltammetry (LSV) from −0.05 to 0.4 V vs RHE at a scan rate of 5 mV s^–1^ in an electrolyte containing 1.0 M KOH and 0.1 M N_2_H_4_ for HzOR activity. HzOR chronoamperometric (CA) tests were carried out on the carbon paper with a size of 1 cm^2^ for 2 h at 0.18 V vs RHE in the same electrolyte, which was replaced every 30 min. The electrode was then measured in the LSV from 1.4 to 1.8 V vs RHE at a scan rate of 5 mV s^–1^ in an electrolyte containing 1.0 M KOH for OER activity and from 0.05 to −0.20 V vs RHE at a scan rate of 5 mV s^–1^ in the same electrolyte for HER activity.

### Electrochemical Surface Area (ECSA) Measurement

The ECSAs of all the electrocatalysts were determined from their CV curves recorded between 0.05 and 1.1 V vs RHE at 50 mV s^–1^ in an Ar-saturated 1.0 M KOH solution. The charges associated with the RhH reduction peak were integrated and used to calculate ECSA according to

$$\mathrm{ECSA}=\frac{Q_{\mathrm{RhH}}}{0{.\mathrm{221 mC cm}}^{-2}\times m_{\mathrm{catalyst}}}$$

where *Q*_RhH_ is the total charges by integrating the desorption peak area of RhH to Rh, *m*_catalyst_ is the mass of the catalyst on the working electrode determined through ICP-MS analysis, and 0.221 mC cm^–2^ is the charges required for the desorption of one RhH monolayer.^30^

## Results and Discussion

Figure 1A illustrates two slightly different protocols that lead to the formation of distinct Rh nanostructures. In the first protocol, aqueous NaBH_4_ is added in one shot into aqueous CTAC preheated to 90 °C, followed by the one-shot injection of aqueous Na_3_RhCl_6_. The reductant preexisting in the reaction solution rapidly converts the incoming Rh­(III) precursor to a large number of Rh atoms, which quickly nucleate and grow into ultrafine nanoparticles and then nanodendrites through attachment growth (Figure 1B). When the sequence of reagent addition is reversed, the added Na_3_RhCl_6_ is quickly diluted by the CTAC solution before NaBH_4_ is added. In this case, the reaction generates quasi-spherical Rh nanoparticles (Figure 1C) due to their major difference in initial reduction rate. In both routes, the two reagents are introduced through one-shot injection with a time interval of about 5 s, minimizing the operational perturbations to the reduction kinetics and growth behavior.

**Figure 1:**
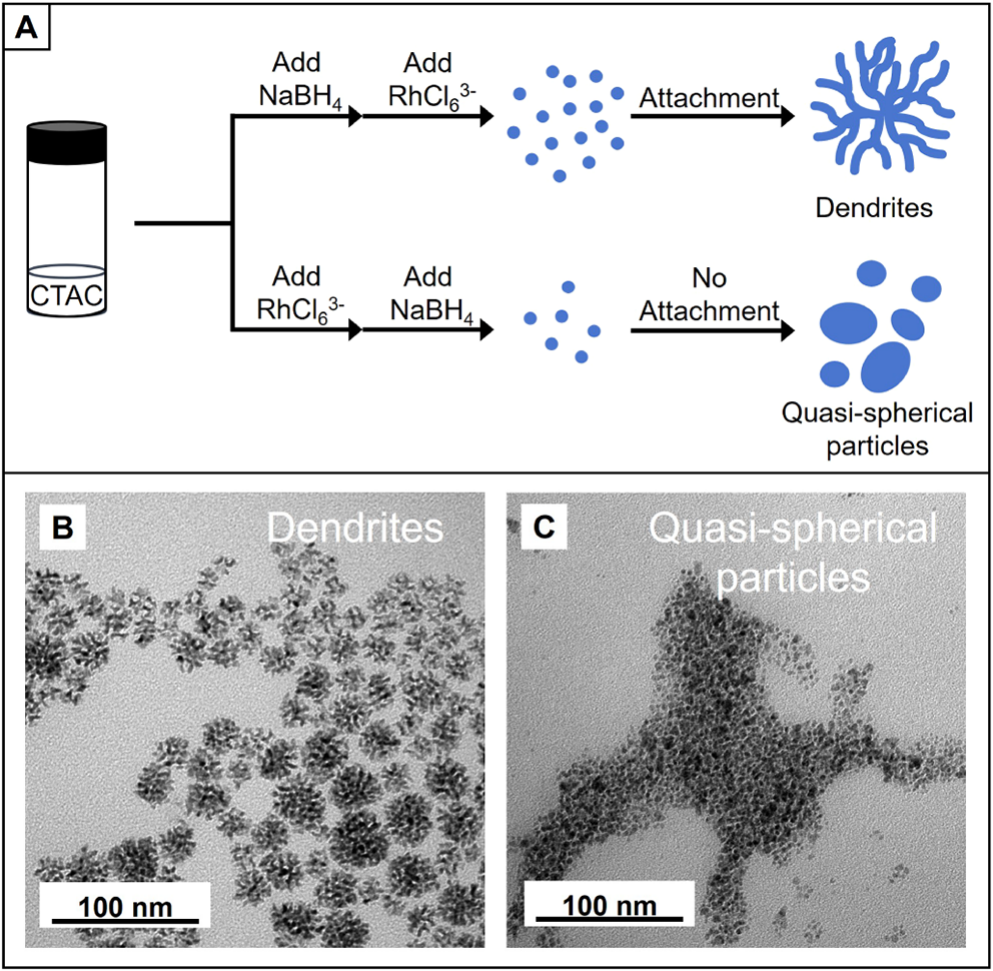
(A) Schematic illustration of two slightly different synthetic protocols leading to distinct nanostructures, (B) TEM image of Rh nanodendrites, and (C) TEM image of quasi-spherical Rh nanoparticles.

As shown by the TEM image in Figure 1B, the Rh nanodendrites displayed a highly branched morphology, together with wavy arms and abundant internal voids, indicative of an attachment-growth pathway. Although there are limited reports on the aqueous synthesis of rhodium nanodendrites, the mechanism underlying the formation of metallic nanodendrites has been extensively explored in the literature.^31^ The reported mechanism is also applicable to current synthesis. Specifically, one-shot injection of Na_3_RhCl_6_ into a solution containing excess NaBH_4_ results in its fast reduction and thus the burst generation of Rh atoms. The transient concentration of Rh atoms becomes much greater than the minimum nucleation concentration, driving extensive self-nucleation for the formation of numerous ultrafine nanoparticles.^32^ These primary particles subsequently attach to each other to form a dendritic framework. Because the majority of Rh atoms are consumed during the rapid nucleation step, their concentration becomes too low to drive appreciable atomic deposition, helping to retain the width of the branched arms. When the sequence of reagent addition is reversed, the Rh precursor is quickly diluted by the CTAC solution, and the maximum attainable concentration of Rh atoms is therefore constrained by this dilution when NaBH_4_ is introduced later. The low transient concentration of Rh atoms reduces the number of self-nucleation events, leaving behind a larger fraction of atoms available for surface deposition through atomic addition. As a result, this protocol favors epitaxial growth on the existing few nuclei, ultimately yielding relatively large quasi-spherical Rh nanoparticles, as shown in Figure 1C.

We also recorded UV–vis spectra from the reaction solution at different time intervals to gain insight into the growth process (Figure S1). During the early stage of syntheses (*t* ≤ 60 s), TEM analysis confirmed that both syntheses yielded ultrafine Rh nuclei with comparable average sizes of about 2 nm (Figure S2). At this size regime, optical extinction is dominated by absorption rather than scattering, and the absorbance can be considered proportional to the number density of nuclei.^33^ Notably, the dendritic system exhibited significantly higher extinction than the quasi-spherical system at this stage, indicating a higher density of primary nuclei due to a much faster nucleation process (Figures S1 and S3). Compared to the continuous increase in extinction observed during the atomic growth of quasi-spherical particles, the dendritic system exhibited a gradual decrease in extinction intensity, indicating that attachment growth reduced the number density of particles in solution through coalescence, despite the enhanced scattering associated with the increase in effective particle size.^34^

High-resolution transmission electron microscopy (HRTEM) was employed to resolve the atomic structures of the nanodendrites. We focused on the representative sample synthesized at 90 °C for 30 min using a freshly prepared Na_3_RhCl_6_ solution (10 mM), which consistently produced well-defined dendritic nanostructures (Figure 2A). We also analyzed the distribution of diameters of the individual branches. Statistical analysis over a large sampling area, including branches from multiple dendrites, revealed an average width of approximately 2.5 nm for the branched arms (Figure 2B). Such ultrathin branches correspond to a high surface-to-volume ratio and the exposure of abundant active sites, making them advantageous for catalytic applications. Selected-area electron diffraction (SAED) was also acquired from the region marked in Figure 2A. As displayed in Figure 2C, the distinct diffraction rings can be indexed to (111), (200), and other planes of face-centered cubic (*fcc*) Rh, confirming that the nanodendrites adopt the expected *fcc* lattice. The continuous ring-like diffraction patterns also indicate the presence of numerous nanocrystallites with variable orientations within the sampled area in accordance with the attachment-growth mechanism, where plenty of primary nanoparticles aggregated in a random orientation manner. X-ray diffraction (XRD) analysis further corroborated the *fcc* structure of Rh (Figure 2D). Notably, a slight shift of the diffraction peaks toward lower angles was observed relative to the standard XRD pattern of *fcc*-Rh, implying the presence of tensile lattice strains in the dendritic nanostructures.

**Figure 2:**
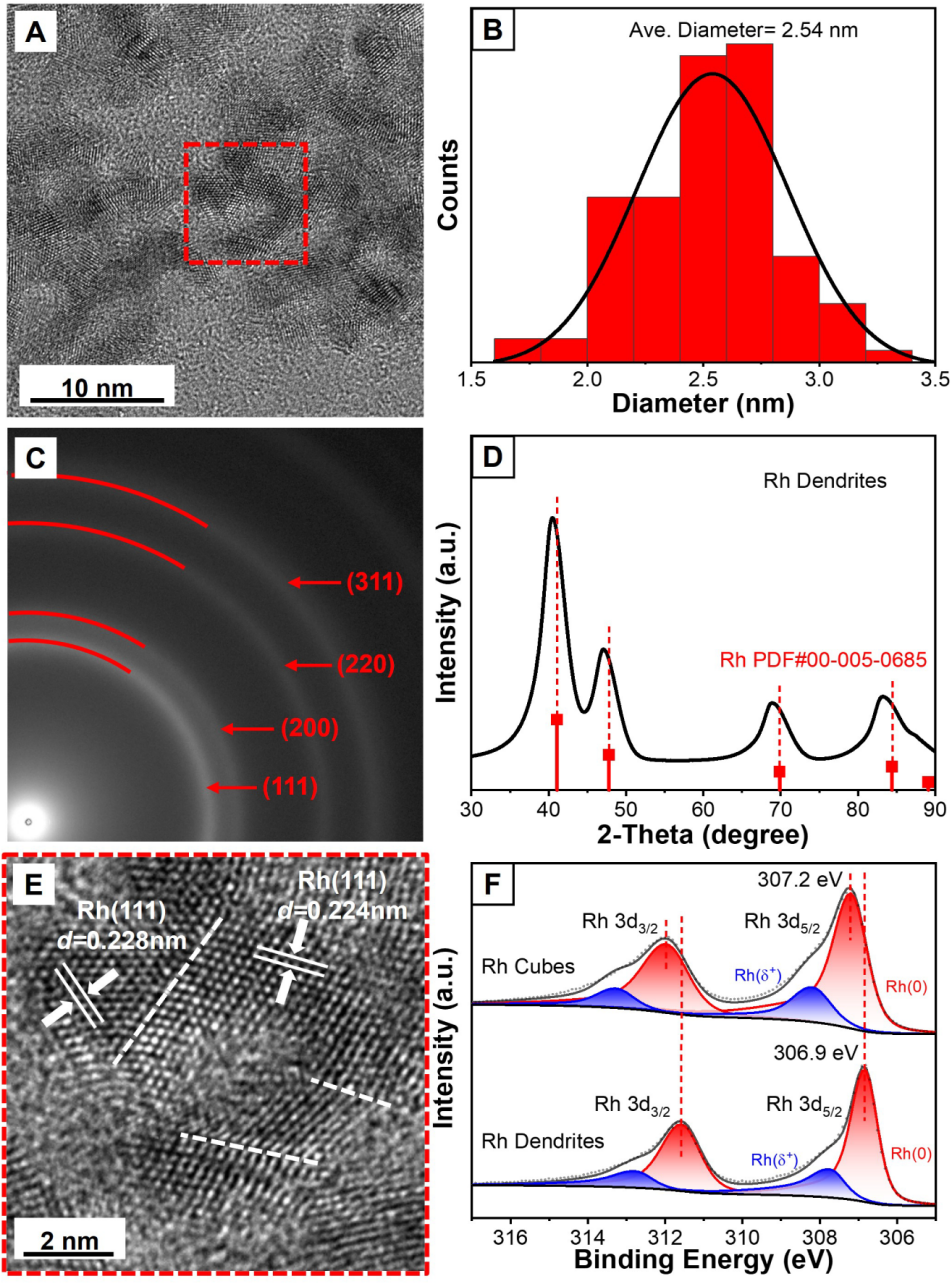
(A) High-resolution TEM image of Rh nanodendrites, (B) diameter distribution of the branched arms, (C) SAED pattern from the same sample in (A), (D) XRD patterns collected from the nanodendrites and standard *fcc*-Rh, (E) tensile lattices and stacking faults on the nanodendrites in the area marked by a red dashed box in (A), and (F) XPS spectra of the nanodendrites and nanocubes.

The enlarged HRTEM image of the branched arms validated the existence of strain (Figure 2E). Plentiful twin boundaries were observed throughout the dendritic frameworks. The lattice spacings in the vicinity of these defects were larger than standard Rh(111) spacing of 0.220 nm. To further obtain lattice spacing statistics at atomic resolution, we analyzed the sample using high-angle annular dark-field scanning transmission electron microscopy (HAADF-STEM, Figure S4) and extracted line-scan profiles from multiple dendritic regions (Figure S5). The statistical results confirmed an expanded interplanar spacing, suggesting the pervasive presence of tensile strains in the dendritic structure (Table S1). Considering the nonequilibrium attachment growth during the formation of Rh nanodendrites, strain states were nonuniformly distributed across different regions rather than showing a homogeneous lattice expansion.^10,11^ X-ray photoelectron spectroscopy (XPS) was also performed to probe the chemical state of Rh nanodendrites and nanocubes (Figure 2F). The Rh(0) 3d_5/2_ peak (306.9 eV) of Rh nanodendrites exhibited a negative shift compared to that of Rh nanocubes (307.2 eV), suggesting that the tensile strain in Rh nanodendrites could tune the electronic structure and increase the electron density around Rh atoms.

To gain a deeper insight into the formation of Rh nanodendrites, we first examined the temporal evolution of their morphology under identical reaction conditions. At a short reaction time (*t* ≤ 60 s), a large population of ultrafine Rh nanoparticles were generated instantaneously, following the rapid color change of the solution from pink to dark brown (Figures S1 and S3). Because attachment growth proceeded at comparatively slower kinetics, the primary nanoparticles had not had enough time to evolve into complete dendritic structures (Figure S2). This observation is consistent with previous reports from our group, where rapid nucleation preceded the onset of attachment-driven construction of nanodendrites.^31^

At *t* = 30 min, well-defined Rh nanodendrites were obtained (Figure 3A). The nanostructure displayed abundant, irregularly oriented branches and a loose, porous framework characteristic of the attachment-growth mechanism. At an extended reaction time, the dendrites continued to grow, and their overall size reached ca. 50 nm at *t* = 60 min (Figure 3C) and ca. 100 nm at *t* = 4 h (Figure 3E). The size distribution also became broader. This trend can be attributed to the fact that larger dendrites possess greater surface areas and thus higher collision probability with the remaining small Rh nanoparticles, causing accelerated particle attachment while promoting size divergence.^35^ Furthermore, as the nanodendrites grow larger, their surfaces exhibit denser, more compact fractal structures with fewer internal voids, limiting the exposure of inner Rh atoms and thereby decreasing the accessible surface area.

**Figure 3:**
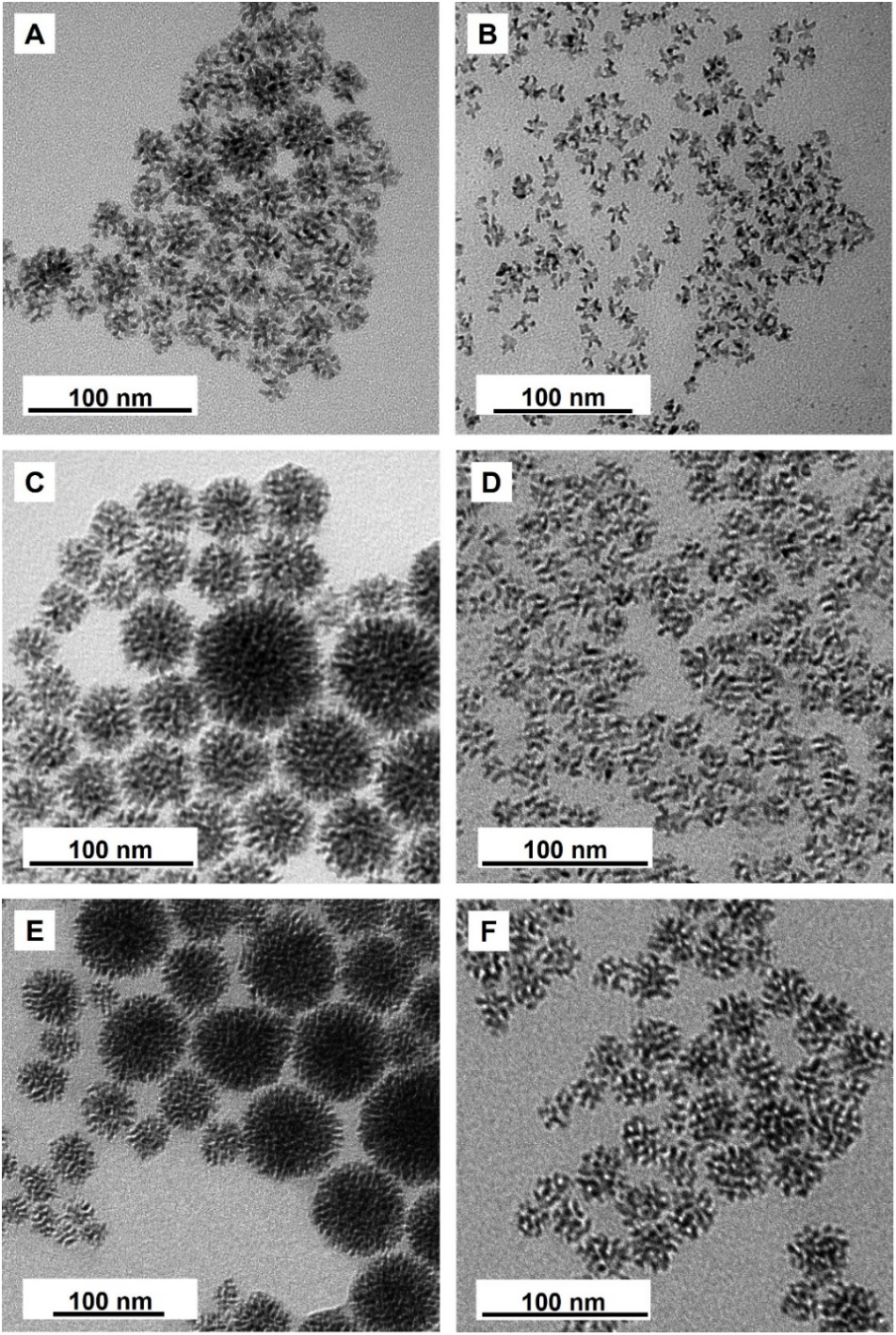
TEM images showing Rh nanodendrites at different time points and with different precursors. Dendritic Rh nanostructures synthesized using freshly prepared RhCl_6_^3–^ precursor at 90 °C for (A) 30 min, (C) 60 min, and (E) 4 h, respectively. Dendritic Rh nanostructures synthesized using aged RhCl_6_^3–^ precursor at 90 °C for (B) 30 min, (D) 60 min, and (F) 4 h, respectively.

To probe the influence of precursor species, we performed parallel experiments using a RhCl_6_^3–^ solution that had been aged for 3 weeks as the precursor. It is known that RhCl_6_^3–^ can undergo ligand exchange and partial hydrolysis in water.^36^ Freshly prepared RhCl_6_^3–^ solutions display a pink color with a characteristic absorption peak at 510 nm, whereas the aged solutions gradually turn yellow and exhibit a blue-shifted absorption peak around 469 nm, indicative of transformation into hydrolyzed species such as Rh­(H_2_O)_*x*_Cl6-_*x*_^*x*^-3 (Figure S6).^37^ Because different coordination ligands alter the reduction kinetics of Rh­(III), the nucleation behavior and subsequent growth mode of the Rh nanoparticles are expected to differ.

Indeed, using the aged precursor, the product obtained at *t* = 30 min (Figure 3B) was found to contain much smaller and more uniform nanodendrites of ca. 10 nm in overall size. This more controlled growth behavior could be attributed to the slower reduction rate of the hydrolyzed precursor species, which decreased the instantaneous concentration of Rh atoms available during nucleation.^38^ Consequently, fewer ultrafine Rh nanoparticles were produced initially, and the dendrites remained in an early stage of attachment growth, exhibiting fewer branches and smaller, more uniform dimensions. As the reaction progressed, the dendrites grew steadily, reaching ca. 25 nm at *t* = 60 min (Figure 3D) and ca. 40 nm at *t* = 4 h (Figure 3F). Notably, even at longer times, their size distribution remained comparatively narrow. This reflects the slower and more uniform attachment process under conditions of a reduced nanoparticle concentration.

Then the influence of precursor concentration was examined. At a low, freshly prepared precursor concentration of 2 mM, only a large population of ultrafine Rh nanoparticles and a few poorly developed dendrites were observed, and the yield was too low to allow for effective isolation. Increasing the precursor concentration to 5 mM enabled recovery of the product and yielded more small dendrites (Figure S7). These results confirm that attachment growth requires a minimum concentration of primary nanoparticles, and insufficient nanoparticle generation at low precursor concentrations fails to sustain dendrite formation, consistent with our mechanistic discussion on the agent addition sequence. The effect of different capping agents was also systematically investigated. Control experiments using CTAB, PVP, and citrate indicated that CTAC provided an optimal balance in stabilizing the ultrafine Rh primary particles and allowing their subsequent attachment growth (Figure S8). Stronger passivation from the Br^–^ ions of CTAB suppressed attachment growth and dendrite formation,^39^ only inducing the generation of ultrafine Rh particles, whereas weaker stabilizers such as PVP or citrate led to overgrowth of primary particles before dendrite formation or irregular aggregation after burst nucleation, respectively.

Temperature also plays a critical role in determining the reduction kinetics, nucleation, and subsequent attachment growth of Rh nanodendrites. Using the aged RhCl_6_^3–^ precursor, we systematically investigated the effect of reaction temperature to elucidate how thermal conditions influence the formation and morphological development of the Rh nanodendrites. At 90 °C, the uniform nanodendrites were obtained (Figure 4A), consistent with the results described earlier. When the reaction temperature was reduced to 60 °C, the number of dendrites decreased markedly, and the products no longer maintained the near-spherical symmetry typically observed for dendritic particles (Figure 4C). This phenomenon can be attributed to two factors: (i) the decreased reduction rate at a lower temperature led to a lower concentration of Rh atoms and consequently fewer primary nanoparticles, and (ii) the substantially weakened thermal motion of small nanoparticles reduced their ability to overcome the energy barrier associated with random attachment, restricting their growth to limited directions that involved lower activation energy.^40^ At room temperature (27 °C), no dendrites were collected and observed (Figure 4E). Although the reaction solution turned light brown, indicating the formation of Rh nanoparticles, the thermal energy was inadequate to drive attachment growth, preventing the formation of dendritic structures.

**Figure 4:**
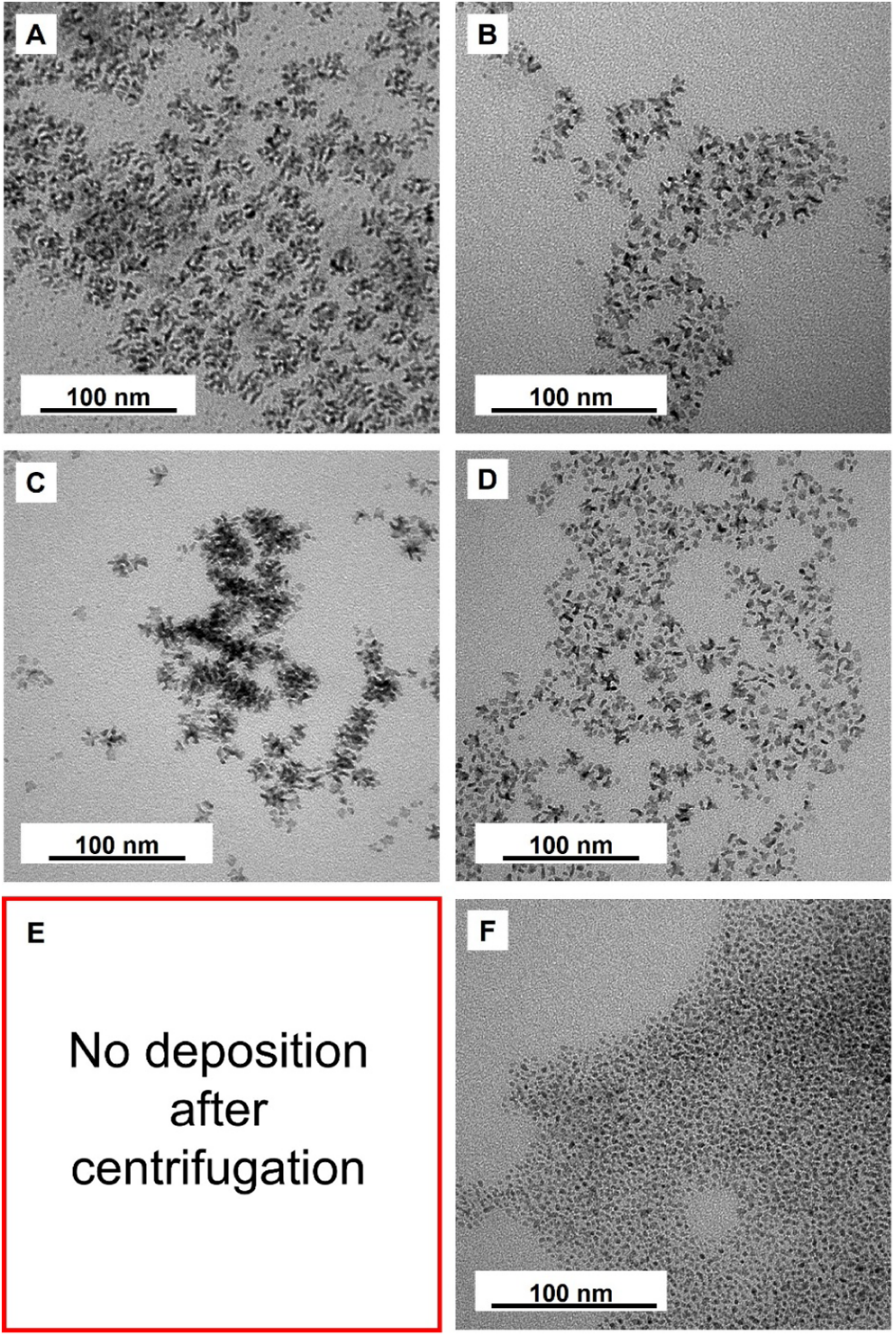
TEM images showing Rh particles obtained at different temperatures. (A) Rh dendrites obtained from aged RhCl_6_^3–^ precursor at 90 °C for 30 min, (B) solid products collected from the supernatant of (A) by reheating at 90 °C for 12 h, (C) solid products obtained from aged RhCl_6_^3–^ precursor at 60 °C for 30 min, (D) solid products collected from the supernatant of (C) by reheating at 90 °C for 12 h, (E) no solid products were obtained from aged RhCl_6_^3–^ precursor at 27 °C for 30 min, and (F) solid products collected from the supernatant of (E) by reheating at 90 °C for 12 h.

To further validate that elevated temperature is essential for initiating attachment growth in this system, the supernatants collected from the syntheses conducted at these three temperatures were reheated to 90 °C for 12 h after centrifugation. For the sample originally synthesized at 90 °C, reheating the supernatant yielded relatively small, early-stage dendrites (Figure 4B). Their reduced size reflects the limited concentration of remaining Rh nanoparticles after the initial synthesis. For the supernatant obtained from the reaction conducted at 60 °C, reheating produced a mixture of small dendrites and numerous partially formed dendritic structures (Figure 4D), consistent with a lower initial nanoparticle concentration while confirming that attachment growth could be reactivated at a sufficiently high temperature.

In contrast, reheating the supernatant from a synthesis conducted at room temperature yielded only irregular Rh particles, with no sign of dendritic growth (Figure 4F). This outcome suggests that at room temperature the reduction kinetics were extremely slow, generating only a small number of nuclei during precursor injection. After dilution, the remaining Rh atoms were insufficient to nucleate and form additional nanoparticles. Instead, the atoms were deposited epitaxially on existing particles, ultimately producing larger, nondendritic Rh nanoparticles that could be isolated by centrifugation. From a mechanistic perspective, the slow reduction at room temperature effectively eliminated the impact of mass transport, resembling the scenario where the precursor was quickly diluted before the introduction of the reductant.

We next evaluated the electrocatalytic performance of the Rh nanodendrites using a rotating disk electrode (RDE) in an alkaline electrolyte solution (1.0 M KOH). As for the catalyst, we focused on the Rh dendrites obtained at *t* = 30 min using the aged precursor solution, together with the 5 nm Rh cubes serving as a benchmark for comparison. The electrochemically active surface area (ECSA) was first determined from cyclic voltammetry (CV) curves acquired in 1.0 M KOH (Figure 5A). The Rh dendrites exhibited a much larger ECSA (177.3 m^2^/g) than the Rh cubes (85.3 m^2^/g), consistent with their ultrathin branches and a highly porous architecture (Figure S9). The differences between the CV peak features of the two catalysts could be attributed to the highly branched morphology and widespread tensile strains present in the dendritic nanostructures. The hydrazine oxidation activity (HzOR) was then assessed in 1.0 M KOH containing 0.1 M N_2_H_4_ (Figure 5B). Remarkably, Rh dendrites delivered a mass activity of 162.0 A mg^–1^ at 0.20 V vs RHE. The required overpotential was only 38 mV at 10 mA cm^–2^, and about 184 mV at 100 mA cm^–2^, representing one of the highest HzOR activities reported to date for a monometallic catalyst. The dendrites also showed excellent hydrogen evolution reaction (HER) performance in 1.0 M KOH (Figure 5C). At −0.07 V vs RHE, they achieved a mass activity of 8.6 A mg^–1^, surpassing the Rh cubes (5.2 A mg^–1^). The good performance toward both HzOR and HER can be attributed to the tensile strains arising from attachment growth, which tuned the local electronic configuration of Rh atoms (Figure 2F), enhanced the adsorption of hydrogen intermediates, and facilitated both anodic and cathodic half-reactions.^41–43^ This dual functionality suggests great potential for the Rh dendrites as bifunctional catalysts.

**Figure 5:**
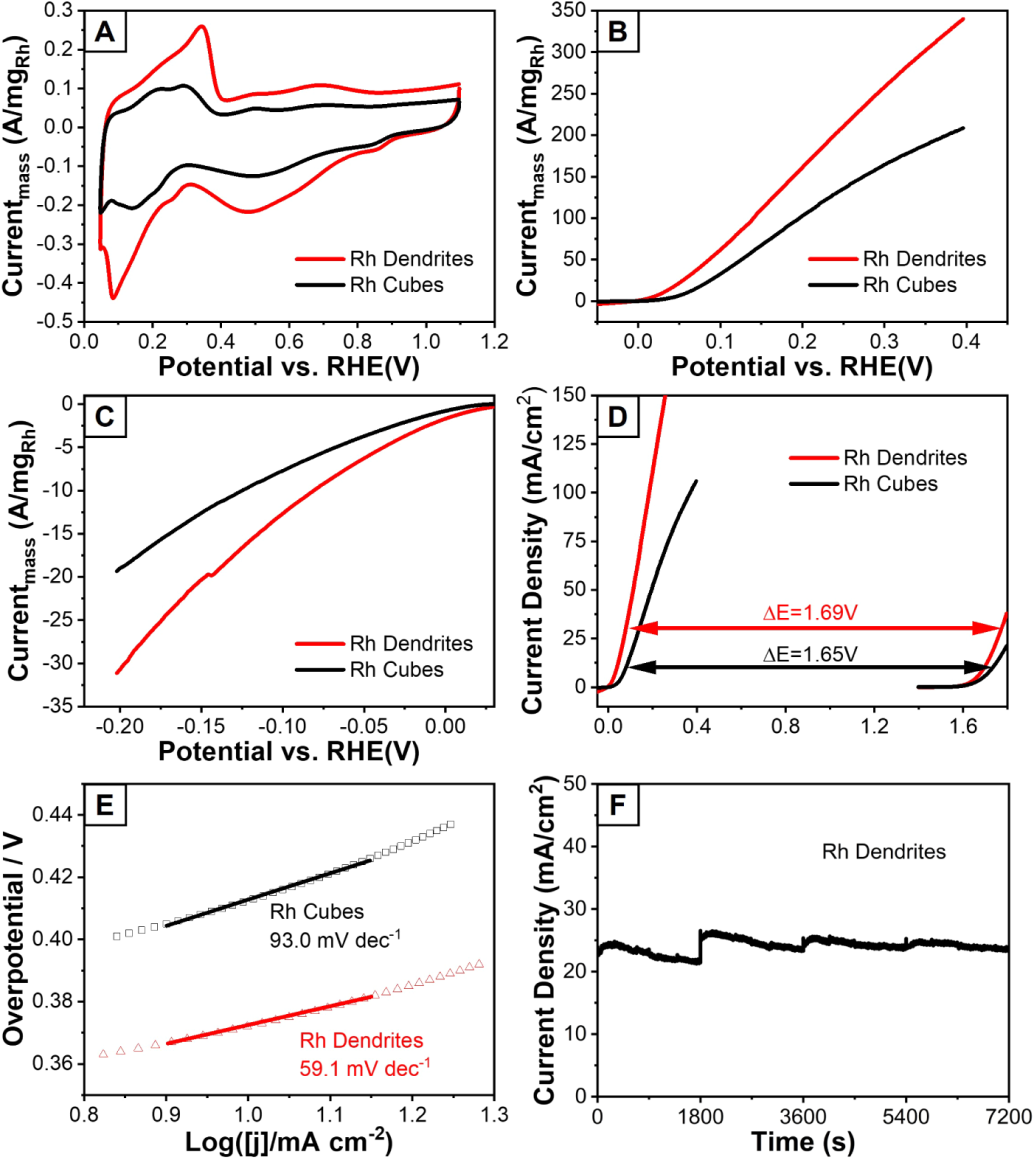
Catalytic performance of the Rh dendrites and cubes. (A) CV curves in 1.0 M KOH electrolyte for ECSA determination, (B) LSV curves for HzOR in a mixture of 0.1 M N_2_H_4_ and 1.0 M KOH, (C) LSV curves for HER in 1.0 M KOH, (D) comparison of the polarization curves for HzOR and OER in a mixture of 0.1 M N_2_H_4_ and 1.0 M KOH, and 1.0 M KOH, respectively, (E) Tafel plots based on LSV curves of HzOR, and (F) chronoamperometric curves in a mixture of 0.1 M N_2_H_4_ and 1.0 M KOH at 0.18 V vs RHE. All LSV tests were conducted at a scan rate of 5 mV s^–1^ and a rotation speed of 1600 rpm.

To illustrate the energetic advantage of hydrazine-assisted water splitting, we compared polarization curves for HzOR and the conventional oxygen evolution reaction (OER) (Figure 5D). Replacing the OER with the HzOR reduced the required cell voltage by approximately 1.69 V, significantly lowering the overall energy consumption while improving hydrogen-generation efficiency. Kinetic analysis further highlights the benefits of the dendritic morphology. The Rh dendrites displayed a smaller Tafel slope (Figure 5E), indicative of more favorable HzOR kinetics compared with the Rh cubes. Long-term stability was assessed through chronoamperometry at 0.18 V vs RHE on the carbon paper. To obtain sufficient Rh dendrites as catalysts, a 10-fold scale-up synthesis was conducted in a three-neck flask, and the dendritic morphology of the product proves the scalability of the protocol (Figure S10). As shown in Figure 5F, the Rh dendrites retained nearly all of the initial activity over a period of 2 h of testing, demonstrating excellent structural stability and catalytic durability.

We also measured the electrocatalytic properties of Rh nanodendrites obtained at *t* = 30 min using the freshly prepared precursor and Rh quasi-spherical nanoparticles obtained at *t* = 12 h (Figures S9 and S11). Compared with the Rh nanodendrites obtained using the aged precursor, the sample synthesized with the freshly prepared precursor showed a smaller ECSA, accompanied by inferior HzOR and HER performance, which can be attributed to a denser morphology and fewer exposed voids between Rh branches. Because of the slow kinetics, the Rh quasi-spherical nanoparticles obtained at *t* = 12 h were selected as the comparison, representing a stage at which epitaxial growth was relatively complete, and their morphology was maintained (Figure S12). The limited performance of rhodium quasi-spherical nanoparticles further highlighted the advantages of the dendritic morphology.

## Conclusions

In summary, we have demonstrated the synthesis of rhodium dendritic nanostructures in an aqueous solution and at a relatively low temperature. By altering the sequence of reagent injection, we can ensure burst nucleation at the moment of precursor addition for the generation of a large number of ultrafine nanoparticles and the subsequent formation of nanodendrites through attachment growth. Atomic-resolution characterizations reveal that the dendrites contain abundant stacking faults originating from the mismatched attachment of nanoparticles. These structural defects induce pronounced tensile strain, leading to expanded lattice spacings throughout the dendritic branches. Our mechanistic study indicates that the formation of Rh nanodendrites requires not only a sufficient supply of primary nanoparticles from burst nucleation but also adequate thermal energy to overcome the energy barrier associated with nanoparticle attachment. Owing to their increased specific surface area and favorable electronic structures caused by tensile strain, the Rh nanodendrites exhibit enhanced activity toward both hydrazine oxidation and hydrogen evolution reactions. Our results highlight the potential of Rh nanodendrites as highly active bifunctional electrocatalysts for hydrazine-assisted hydrogen production.

## Supplementary Material



## Data Availability

All data supporting the findings of this study are available within the main text, figures, and Supporting Information, or from the corresponding author upon request. Source data are provided with this paper.

## Notes

Additional UV–vis spectra, TEM and STEM images, and electrochemical performance data for Rh dendrites and other nanoparticles (PDF)
