# Supplementary material for: Facile Synthesis of Rhodium Nanodendrites with Enhanced Activity toward Hydrazine-Assisted Water Splitting
Source: Chem Mater. 2026 Apr 21;38(9):4659–67. doi: 10.1021/acs.chemmater.6c00125 (PMC13173500; doi:10.1021/acs.chemmater.6c00125)
Supplement: Supplementary file 1 [file cm6c00125_si_001.pdf]

Supplementary Information

Facile Synthesis of Rhodium Nanodendrites with Enhanced Activity toward Hydrazine-Assisted Water Splitting

Jiaqi Guan,[†] Zhiqi Wang,[†] Kei Kwan Li,[†] Yong Ding,[§] and Younan Xia[†,¶,€,*]

[†]School of Chemistry and Biochemistry, Georgia Institute of Technology, Atlanta, Georgia 30332, United States

[§]School of Materials Science and Engineering, Georgia Institute of Technology, Atlanta, Georgia 30332, United States

[¶]The Wallace H. Coulter Department of Biomedical Engineering, Georgia Institute of Technology and Emory University, Atlanta, Georgia 30332, United States

[€]Department of Materials Science and Engineering, Department of Biomedical Engineering, Johns Hopkins University, Baltimore, Maryland 21218, United States

*Corresponding author. E-mail: yxia70@jh.edu

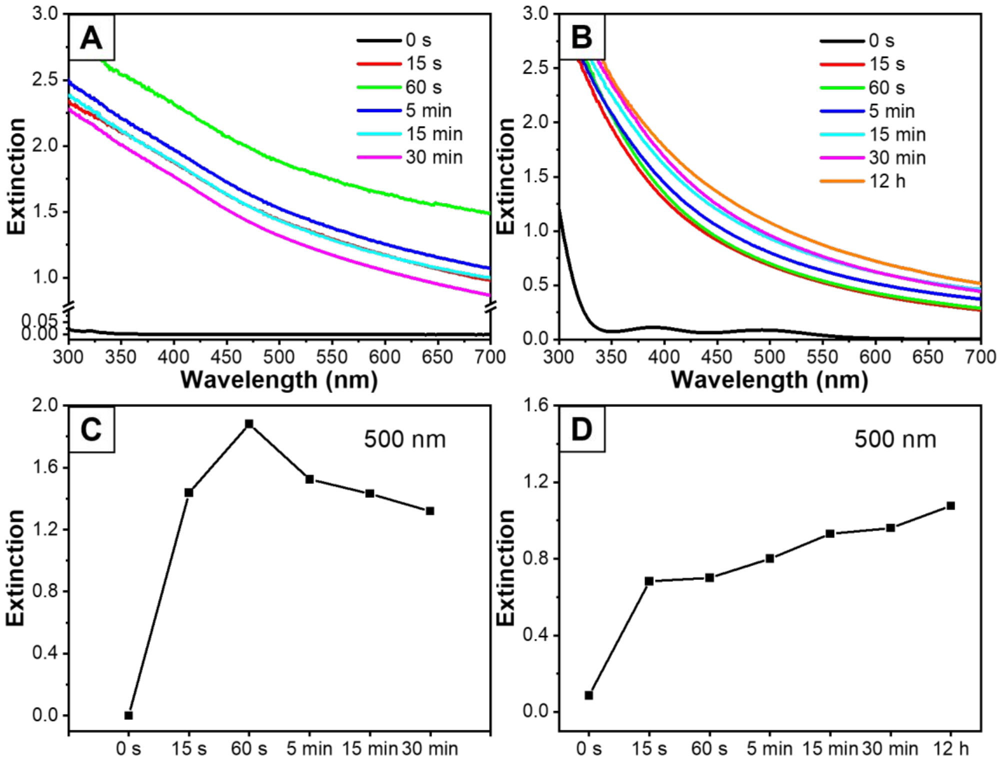

Figure S1. Evolution of UV-vis spectra from the reaction solution for the syntheses of (A) Rh nanodendrites and (B) Rh quasi-spherical particles. Comparison of absorbance at different time intervals of (C) Rh nanodendrites and (D) Rh quasi-spherical particles at 500 nm. At t = 0 s, the reaction solution of (A) included CTAC and $NaBH_4$, and the reaction solution of (B) included CTAC and Na_3RhCl_6. The obtained absorbance values correspond to overall optical extinction (absorption + scattering).

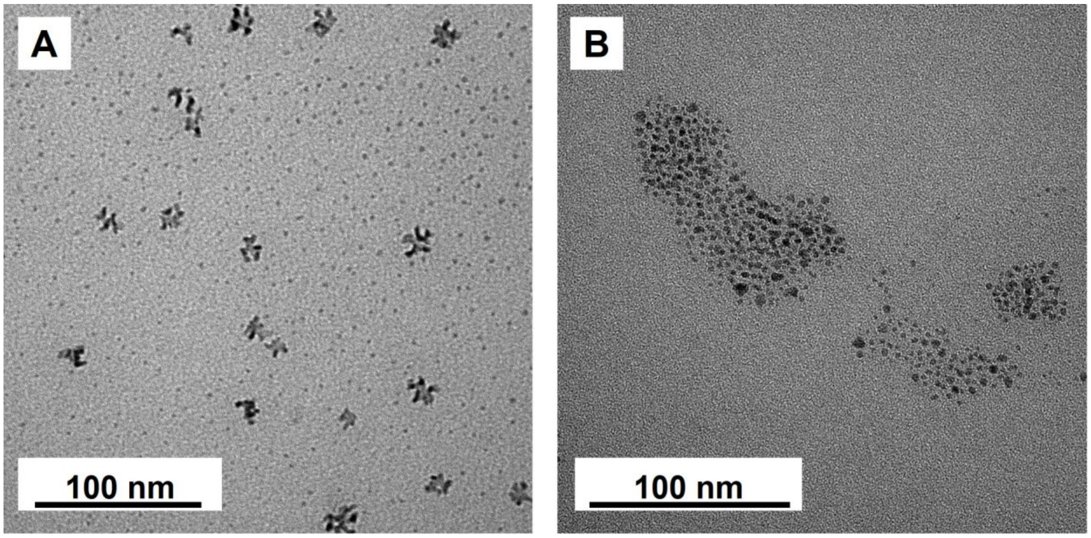

Figure S2. TEM images of the samples obtained in the nucleation stage (t = 60 s) of (A) Rh nanodendrites and (B) Rh quasi-spherical particles.

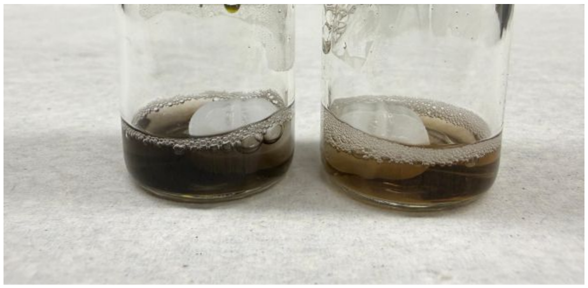

Figure S3. Photo recorded in the nucleation stage (t = 60 s) of (left) Rh nanodendrites and (right) Rh quasi-spherical particles.

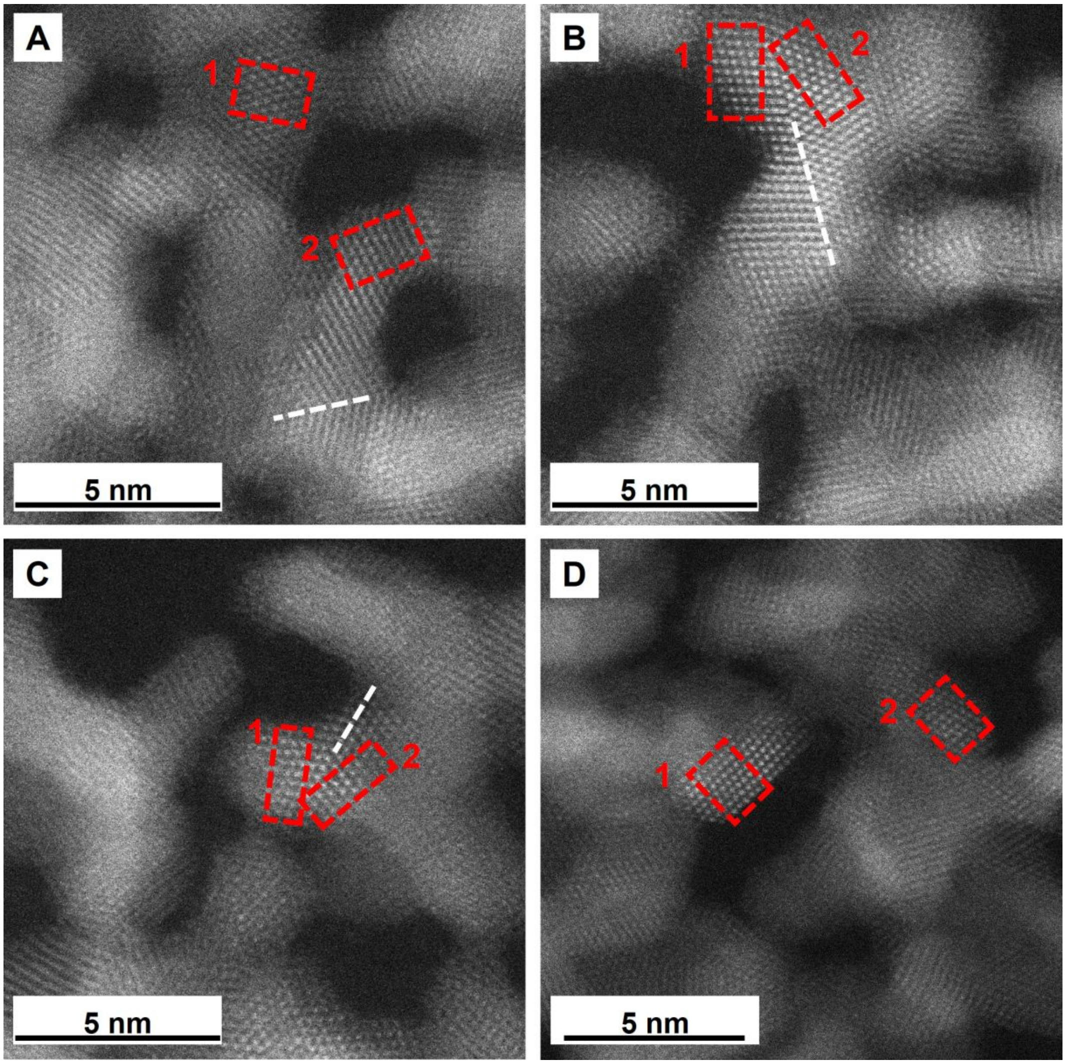

Figure S4. STEM images of Rh dendrites. Tensile lattices and stacking faults on the nanodendrites are marked by a red dashed box and white dashed line.

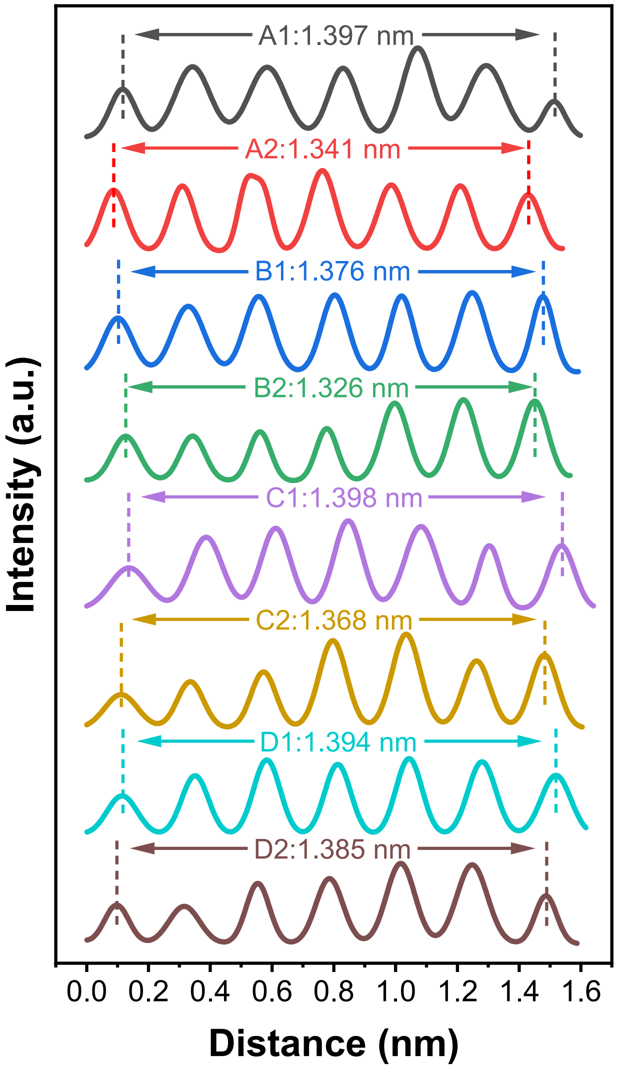

Figure S5. Line profiles extracted from the red dashed regions in Figure S4 (A-D correspond to the respective STEM images, while 1 and 2 denote line profiles obtained from two different selected regions within each image). The measured distances represent the total lattice spacing across seven atomic columns.

Table S1. Average lattice spacings in different red dashed regions.

	A	B	C	D
1	0.233 nm	0.229 nm	0.233 nm	0.232 nm
2	0.224 nm	0.221 nm	0.228 nm	0.231 nm

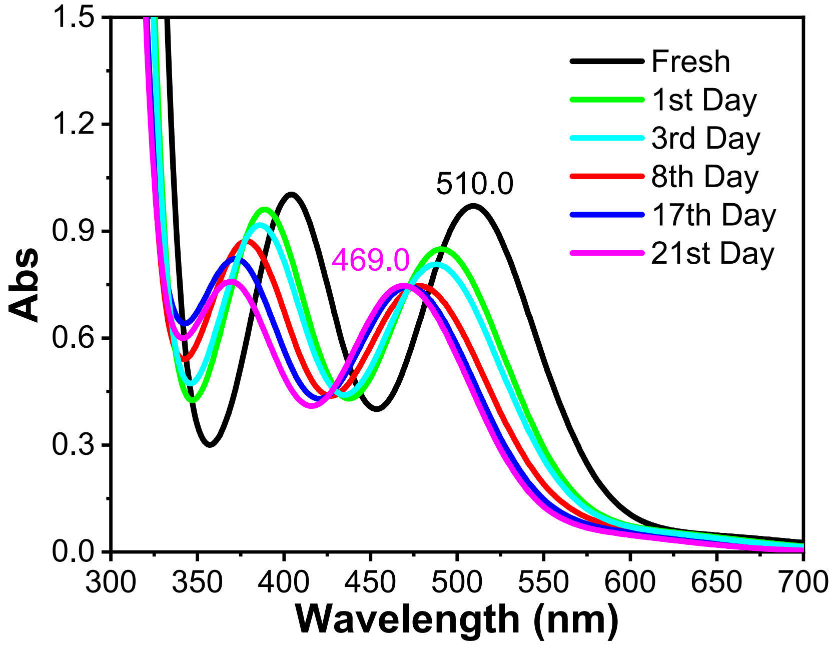

Figure S6. Evolution of UV-vis spectra of $RhCl_6^{3-}$ solutions (10.0 mM) that served as the Rh(III) precursor during the aging process.

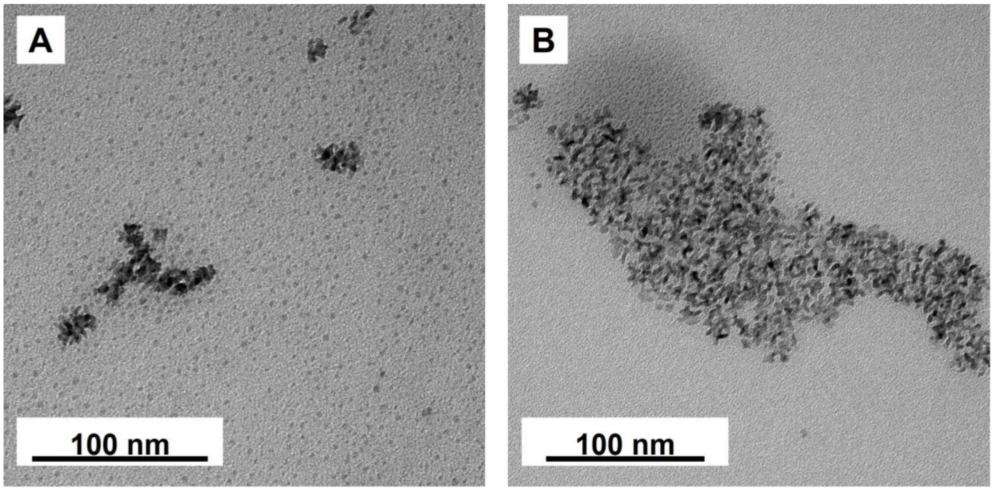

Figure S7. TEM images of Rh nanodendrites synthesized at 90 °C for 30 min using freshly prepared $RhCl_6^{3-}$ precursors at concentrations of (A) 2.0 mM and (B) 5.0 mM.

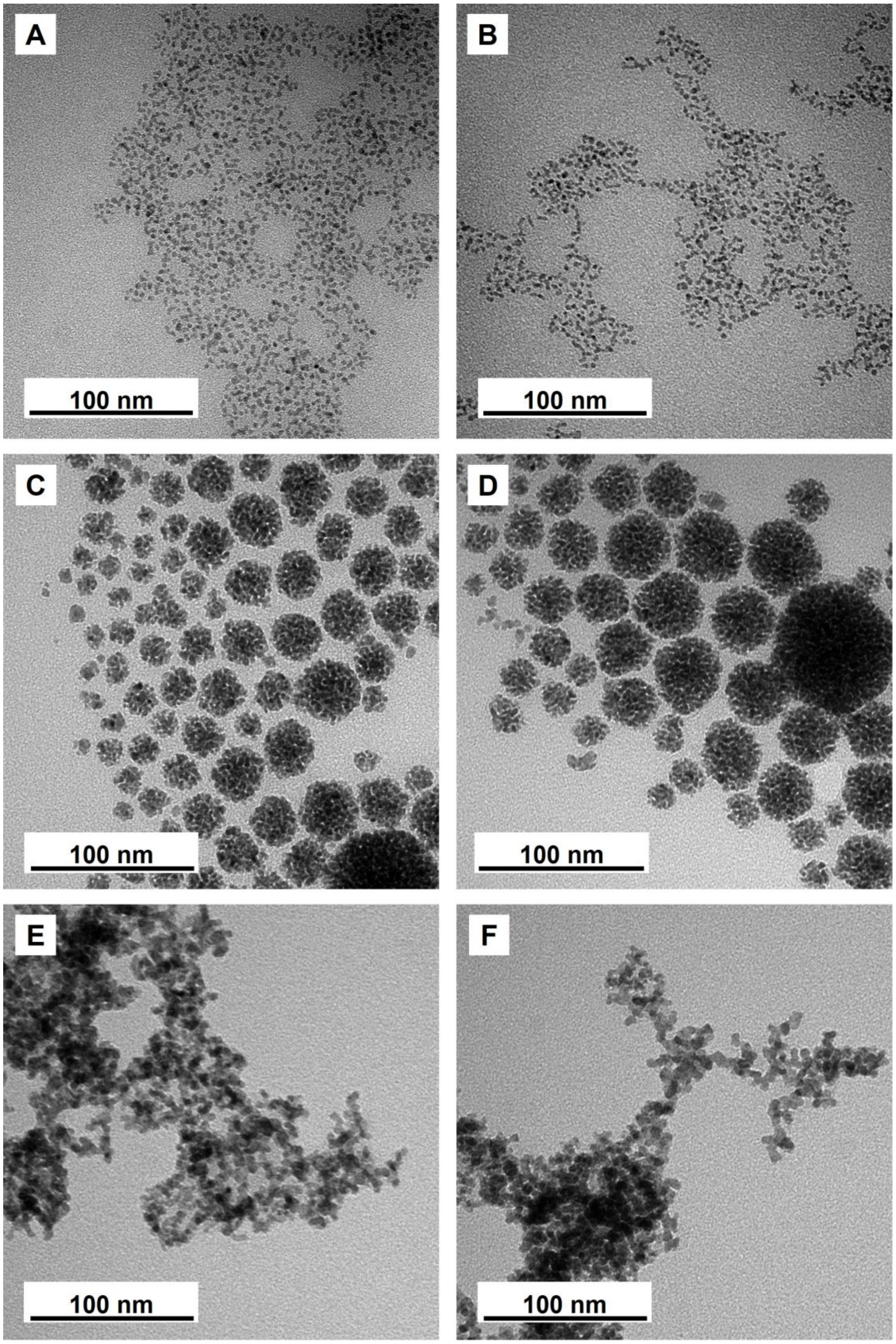

Figure S8. TEM images of Rh nanoparticles synthesized at 90 °C for 30 min using (A) CTAB (100 mM), (B) CTAB (200 mM), (C) PVP (5 mg/ml), (D) PVP (10 mg/ml), (E) citrate (30 mM) and (F) citrate (50 mM) as the capping agents.

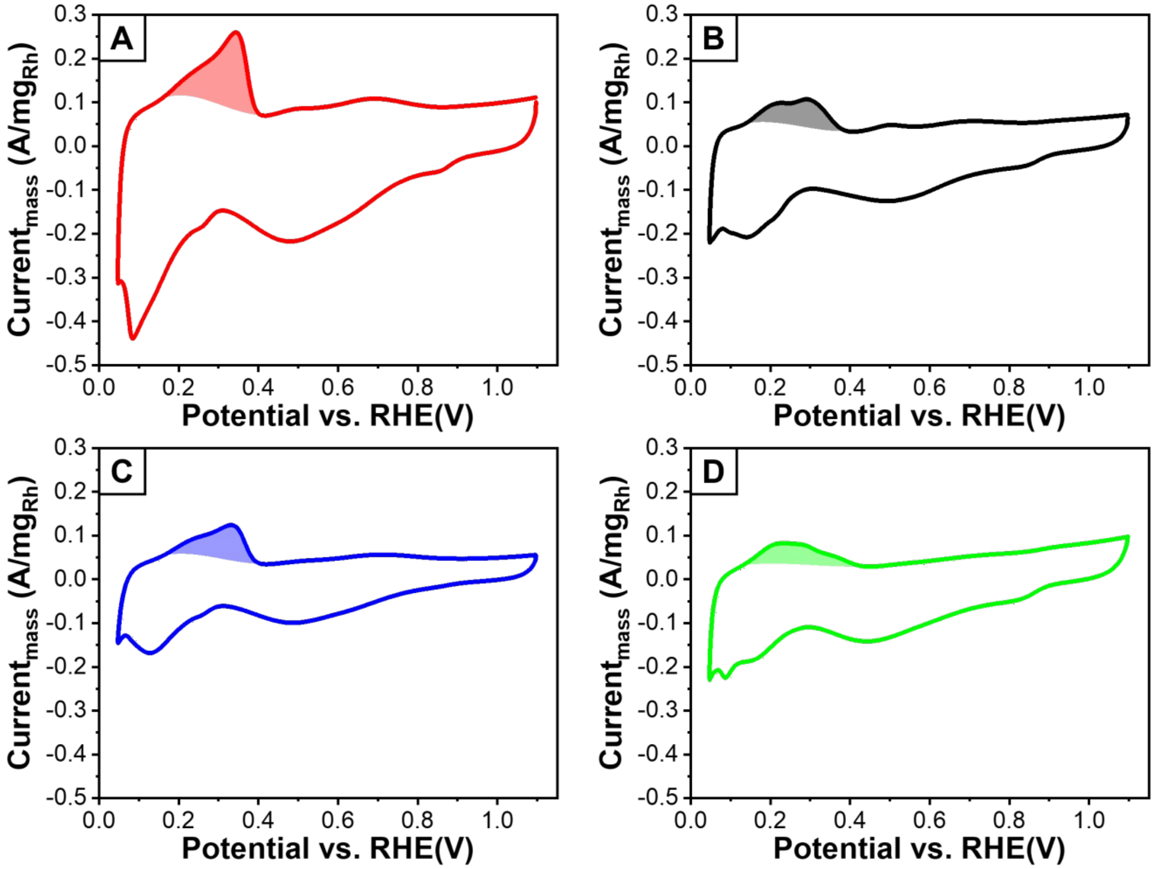

Figure S9. ECSAs measured using the H_{UPD} method. CV curves of (A) Rh dendrites synthesized using aged Rh(III) precursor, (B) Rh cubes, (C) Rh dendrites synthesized using fresh Rh(III) precursor, and (D) Rh quasi-shperical particles recorded in 1.0 M KOH solution, normalized to the mass of the sample.

Table S2. ECSAs of Rh dendrites synthesized using aged Rh(III) precursor, Rh cubes, Rh dendrites synthesized using fresh Rh(III) precursor, and Rh quasi-spherical particles.

Sample name	Rh dendrites (aged precursor)	Rh cubes	Rh dendrites (fresh precursor)	Rh quasi-shperes
ECSA (m^2 g^{-1})	177.3	85.3	89.5	79.9

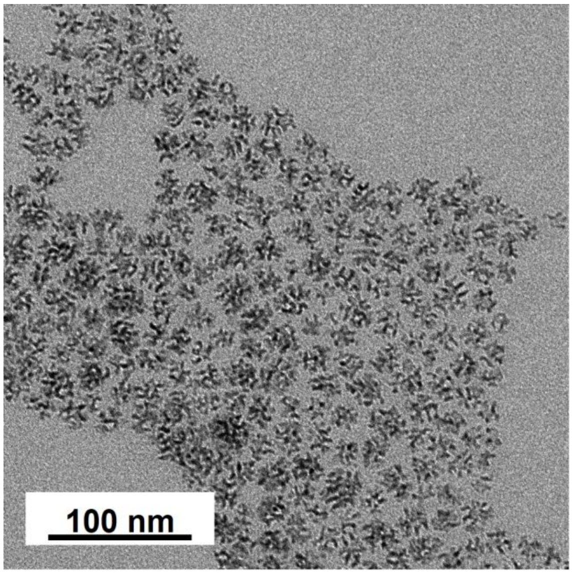

Figure S10. TEM image of Rh nanodendrites synthesized via a tenfold scale-up process in a three-neck flask at 90 °C for 30 min.

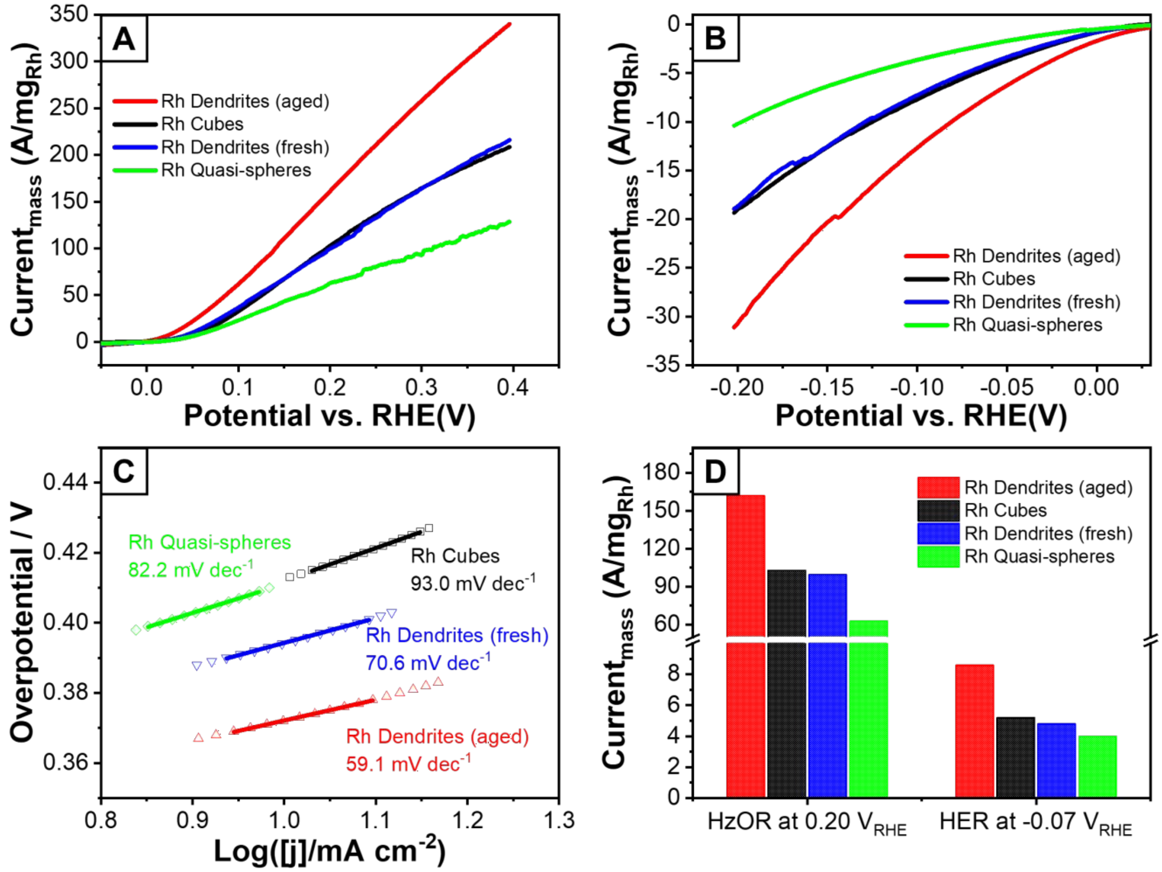

Figure S11. Catalytic performance of Rh nanodendrites synthesized using aged or fresh Rh(III) precursor, quasi-spherical nanoparticles, and nanocubes. (A) LSV curves for HzOR in a mixture of 0.1 M N_2H_4 and 1.0 M KOH, (B) LSV curves for HER in 1.0 M KOH, (C) Tafel plots based on LSV curves of HzOR, and (D) mass activities of the catalysts based on HzOR and HER at the specific potentials.

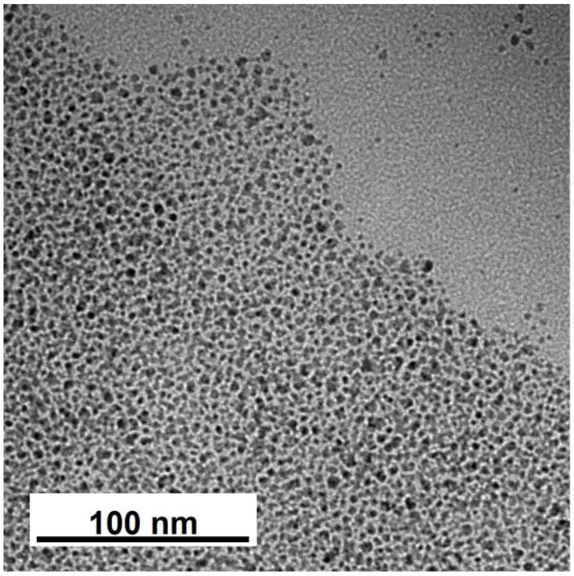

Figure S12. TEM image of Rh quasi-spherical nanoparticles synthesized at 90 °C for 12 h.
